# Automatic Detection of Gastric Wall Structure Based on Oral Contrast-Enhanced Ultrasound and Its Application on Tumor Screening

**DOI:** 10.3389/fonc.2021.627556

**Published:** 2021-03-29

**Authors:** An Sui, Zhaoyu Hu, Xuan Xie, Yinhui Deng, Yuanyuan Wang, Jinhua Yu, Li Shen

**Affiliations:** ^1^Electronic Engineering Department, Fudan University, Shanghai, China; ^2^Department of Ultrasound, Chongming Branch, Xinhua Hospital Affiliated to Shanghai Jiaotong University School of Medicine, Shanghai, China

**Keywords:** gastric cancer, ultrasound, U-net, anisotropic diffusion, edge detection

## Abstract

Gastric cancer is the second most lethal type of malignant tumor in the world. Early diagnosis of gastric cancer can reduce the transformation to advanced cancer and improve the early treatment rate. As a cheap, real-time, non-invasive examination method, oral contrast-enhanced ultrasonography (OCUS) is a more acceptable way to diagnose gastric cancer than interventional diagnostic methods such as gastroscopy. In this paper, we proposed a new method for the diagnosis of gastric diseases by automatically analyzing the hierarchical structure of gastric wall in gastric ultrasound images, which is helpful to quantify the diagnosis information of gastric diseases and is a useful attempt for early screening of gastric cancer. We designed a gastric wall detection network based on U-net. On this basis, anisotropic diffusion technology was used to extract the layered structure of the gastric wall. A simple and useful gastric cancer screening model was obtained by calculating and counting the thickness of the five-layer structure of the gastric wall. The experimental results showed that our model can accurately identify the gastric wall, and it was found that the layered parameters of abnormal gastric wall is significantly different from that of normal gastric wall. For the screening of gastric disease, a statistical model based on gastric wall stratification can give a screening accuracy of 95% with AUC of 0.92.

## Introduction

Gastric cancer is one of the common malignant tumors. The incidence and mortality of gastric cancer in China account for almost half of the world’s annual rate (1). The prognosis of gastric cancer is closely related to the timing of diagnosis and treatment. The 5-year survival rate of patients with advanced gastric cancer is still less than 30% even if they receive comprehensive treatment mainly by surgery (2–5). Early diagnosis of gastric cancer can make the clinical stage of the tumor move forward, reduce the transformation to advanced cancer, improve the early treatment rate and the overall cure rate of gastric cancer, which can not only save but also improve the consumption quality of medical resources (6). Therefore, early diagnosis and treatment of gastric cancer has great clinical value.

At present, the medical imaging methods used in the diagnosis of gastric cancer mainly include gastroscopy, CT, MRI and gastric ultrasound. Histopathological diagnosis of gastric mucosa biopsy under gastroscope is the gold standard for the diagnosis of gastric cancer. Gastroscopy and biopsy of gastric mucosa are highly valued and recommended all over the world (7). However, the early diagnostic rate of gastric cancer in developing countries is still unsatisfactory. In China, the early diagnosis and treatment rate of gastric cancer is only about 10% (8). Moreover, as an invasive examination method, gastroscopy has poor acceptability in the population and is difficult to be popularized as a screening method for gastric cancer. CT has high spatial resolution and clear anatomical structure, which is an important examination method for gastric diseases. But the ionizing radiation of CT is harmful to human body. In addition to the long scanning time and expensive price, MRI is also easy to be affected by the difference of pre-scanning disposition and type, field strength, sequence and parameters, which leads to the unsatisfactory imaging stability of MRI in gastric cancer and cannot be widely used in clinical screening.

In recent years, the application of gastric ultrasound is becoming more and more popular, which has unique advantages. Ultrasound imaging is non-invasive, painless, cheap, convenient and real-time. Because the ultrasound beam can penetrate the gastric wall and display the various levels of gastric wall structure, gastric ultrasound has great application value in the diagnosis of gastric diseases, and has a higher detection rate of gastric wall thickening lesions. As a non-invasive and efficient diagnostic method, gastric ultrasound can provide clinicians with a lot of valuable information, timely detect the changes of gastric wall in terms of morphology and thickness, help to estimate the extent of invasion of gastric wall and understand the metastasis and diffusion of various organs around the stomach (9). And it has been preliminarily proved that trans-abdominal ultrasonography can detect gastric cancer early from histopathology and ultrasound physical characteristics (10–12).

In this paper, we proposed a new method based on U-net to automatically identify the gastric wall area in the gastric ultrasound image. The anisotropic diffusion filter and edge detection method are used to stratify the gastric wall structure and calculate the ratio of each layer, which can be used as a reference to diagnose the disease. It’s a preliminary report on diagnosis of gastric diseases by using the ratio of thickness of each layer of gastric wall. It is helpful to quantify the diagnostic information of gastric diseases, improve the accuracy of ultrasound diagnosis of gastric cancer, and is expected to improve the screening efficiency of gastric cancer.

## Materials and Methods

### Materials

In this study, we collected 251 gastric ultrasound images from 106 patients, including 47 male patients and 59 female patients. 32 cases were diagnosed with gastric disease, 11 cases were diagnosed with gastric cancer, and the rest of cases are normal. Gastric diseases included 10 cases of gastric ulcer, 12 cases of chronic gastritis, 8 cases of acute gastritis, 1 case of gastric stromal tumor and 1 case of gastric polyps. And the sites of diseases included gastric body, gastric antrum and gastric horn. In 11 cases of gastric cancer, there were 9 cases of early gastric cancer and 2 cases of advanced gastric cancer. For the cases of early gastric cancer, the sites of canceration included gastric body and gastric antrum. One case of gastric body cancer and one case of gastric antrum cancer were pathologically diagnosed as intramucosal cancer, and the other 7 cases were adenocarcinoma. The other two cases of advanced gastric cancer were gastric cardia cancer and gastric body cancer, and both were pathologically diagnosed as adenocarcinoma. The patient characteristics of three cohorts are summarized in **Table 1**. It is worth pointing out that these 11 cases of gastric cancer were found to be abnormal by ultrasound examination for the first time, and they were finally confirmed to be gastric cancer through surgery and pathology.

**Table 1:** Patient characteristics of three cohorts.

Characteristics	Normal Cohort	Benign Lesions Cohort	Gastric Cancer Cohort
Age (Mean ± SD)	53.04 ± 14.96	58.04 ± 18.06	71.43 ± 9.33
Sex			
Male	7	8	5
Female	16	16	2
Total	23	24	7

### Ultrasound Scanning Method

The scanning equipment we used is WISONIC Clover 60 portable color Doppler ultrasound diagnostic instrument (Huasheng Medical Technology Co., Ltd, Shenzhen, China) and Ge LOGIQ E9 (GE company, America). Convex array probes are routinely used with a frequency of 5.0MHz. The center frequency will be appropriately adjusted according to the weight of the patient. The patient obeyed the requirement and fasted for 8 hours before the examination to ensure the gastric cavity was empty. During the examination, the patient drinks 500-700 mL warm water with 48g of ultrasonic contrast agent (Xin Zhang^®^, Huqingyutang Pharmaceutical Company, Hangzhou, China). The stomach body, gastric angle, gastric antrum, pylorus and duodenal bulb were routinely screened when patients are in the standing position, and the cardia and gastric fundus were checked in the supine position. If necessary, take the left, right and semi-recumbent positions as a supplementary examination position. The examiner observes the cardia, the fundus of the stomach, the body of the stomach, the corner of the stomach, the antrum, and the pylorus in turn. If gastric lesions are found, perform local image magnification or use high-frequency probes to observe the hierarchical structure of the stomach wall, the shape of the lesion, size range and its relationship with neighboring organs, etc.

To be specific, scanning can be divided into five steps:

1. Scan the cardia, ask the patient to lie on his back and move the probe from xiphoid process to the left costal arch.
2. The gastric fundus is scanned by placing the probe in the 10th intercostal space.
3. Scan the cross section of gastric fundus, body and antrum, ask the patient to lie on the right side, and move the probe from the left costal arch along the contour of the stomach.
4. Scan the coronal plane of gastric fundus, body and antrum, ask the patient to lie on the right side, take the probe tail as the fulcrum, rotate the probe along the left rib arch, and tilt the probe 45° at the same time.
5. Scan the gastric antrum and pylorus, ask the patient to lie on his back, and place the probe at the right vertical costal arch.

### Methods

**Figure 1:**
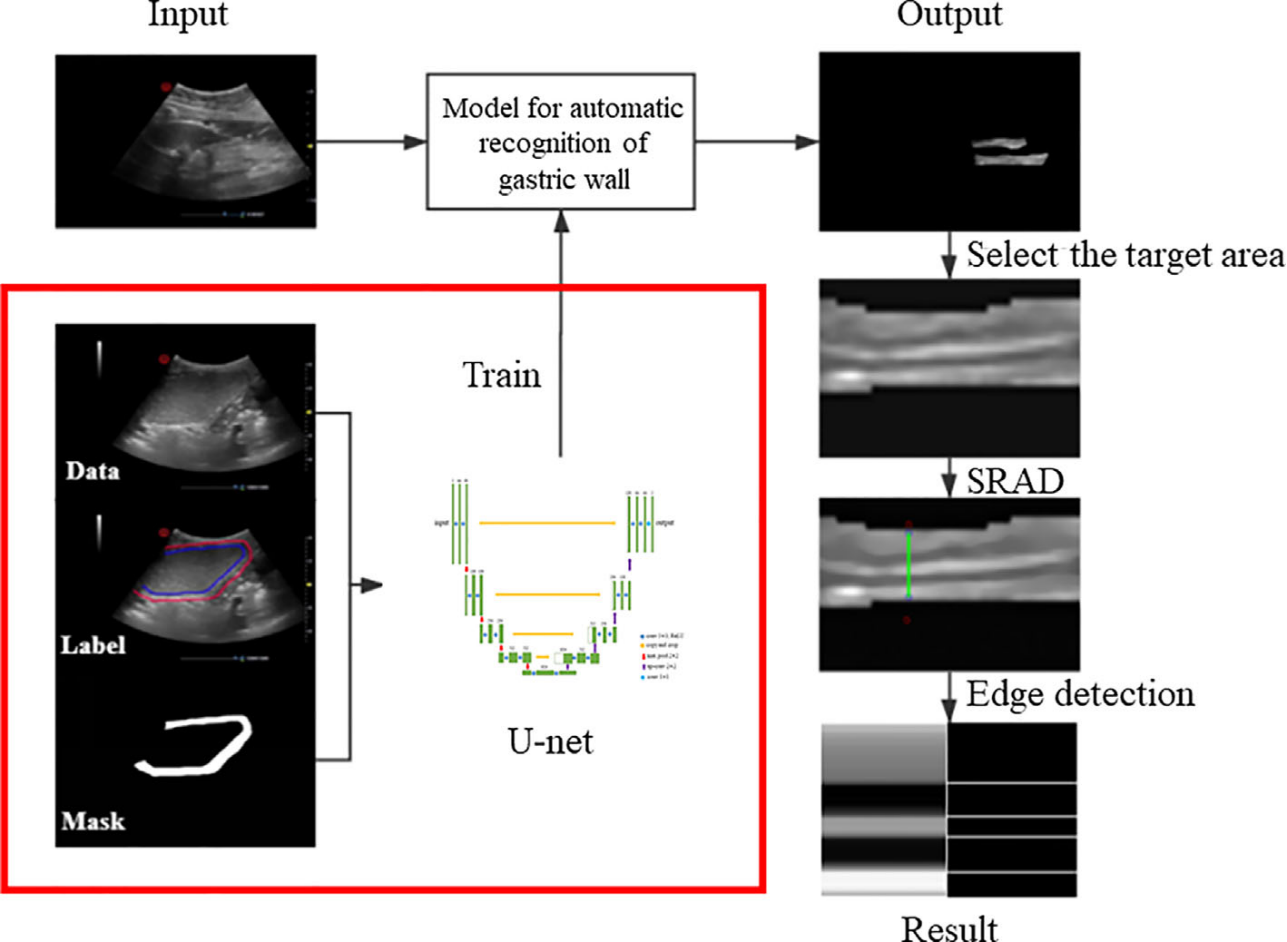
Flow chart of the method.

The flow chart of the method is shown in **Figure 1**. First, a U-net-based gastric wall detection network is used to detect the region-of-interest (ROI) area of the gastric wall. Then, in the detected ROI area, anisotropic diffusion technology is used to extract the layered structure of the gastric wall. By calculating and counting the thickness of the five-layer structure of the gastric wall, a simple and useful gastric cancer screening model was obtained.

We labeled the regions of gastric wall in 251 gastric ultrasound images, and generated a mask corresponding to every single data as the input of U-net. After training, we will get a model which can automatically identify the ROI of gastric wall.

**Figure 2:**
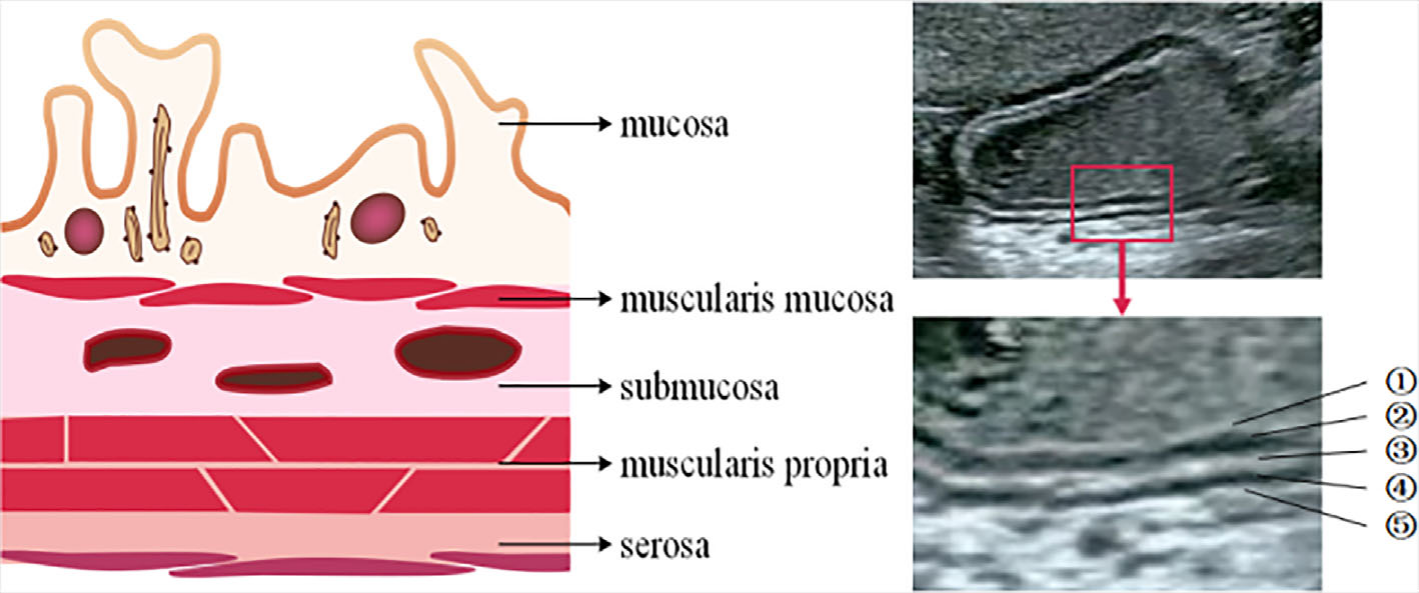
The structure of gastric wall. The gastric wall has five layers.

The gastric wall has five layers, which are mucosa, muscularis mucosa, submucosa, muscularis propria and serosa. The change of thickness ratio of each layer can be used as the basis for diagnosing gastric diseases (**Figure 2**).

The image output from the model is filtered by speckle reduced anisotropic diffusion (SRAD) to make the hierarchical structure of gastric wall more obvious. The edge detection algorithm is used to find the four boundaries among the five levels, and then the five-layer structure is obtained. The ratio of thickness between layers will be calculated, and we can distinguish them by comparing the result of normal gastric wall with that of abnormal gastric wall.

#### Gastric Wall Detection Based on U-Net

**Figure 3:**
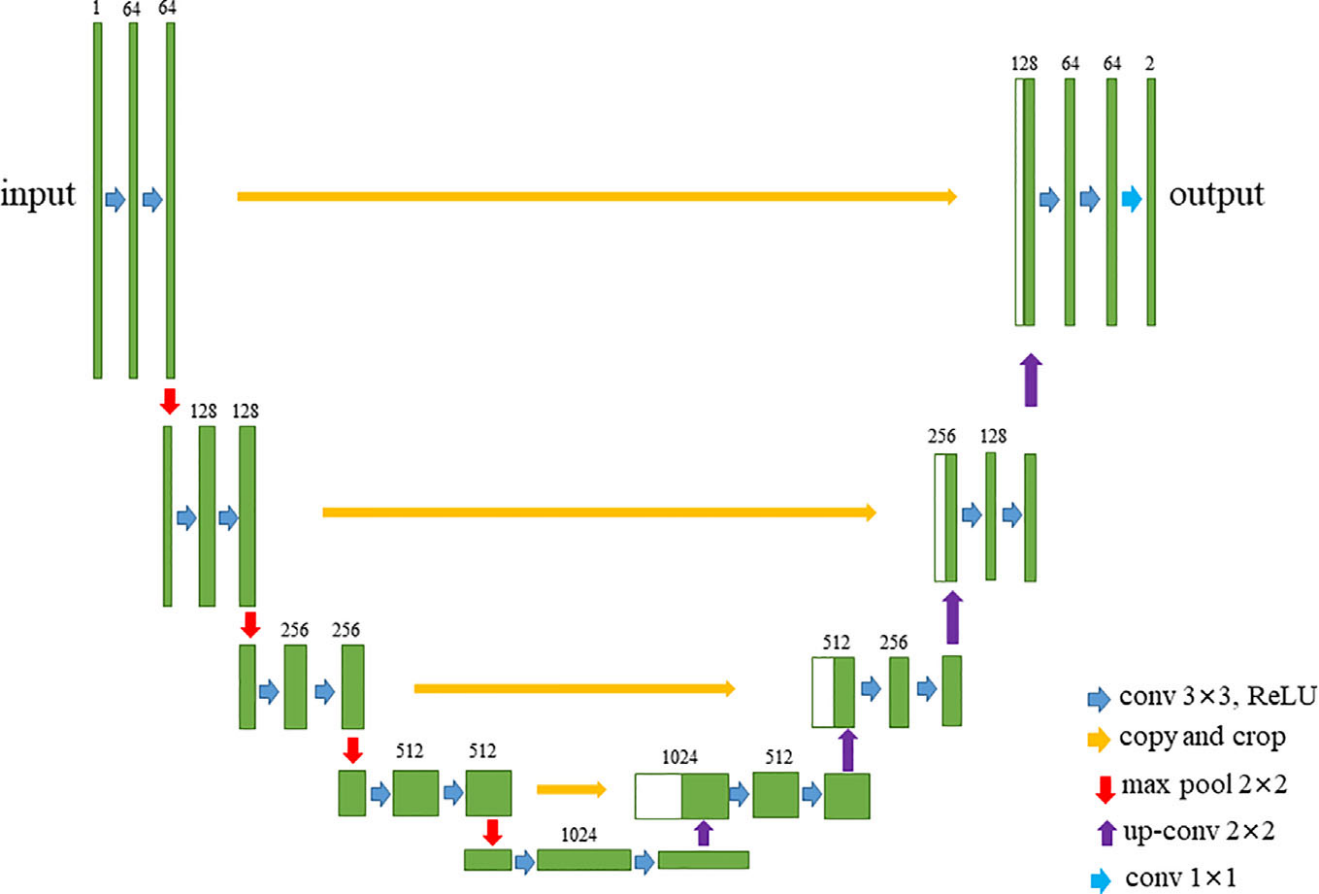
The structure of U-Net.

Because the structure of the stomach is fixed and the semantic information is not rich, it is basically the stomach cavity and the stomach wall, so it is necessary to refer to the high-level semantic information and the low-level semantic information in the work of automatic identification of gastric wall. In addition, the data acquisition of medical images is much more difficult than other images, so the model we designed should not be too large because of the small amount of data. Otherwise, too many parameters will easily lead to over fitting and poor prediction effect. Based on the above reasons, we chose U-net structure to establish the model of automatic identification of gastric wall (**Figure 3**). It can meet the needs of referencing low-level and high-level semantic information at the same time, and solve the problem that the amount of data is small and it is difficult to build an accurate model.

U-net consists of two paths, the contraction path on the left and the expansion path on the right. In the contraction path, there are 10 times of 3 × 3 convolutions (the size of convolution kernel is 3 × 3), the relu activation layer, and four times of 2 × 2 max pooling. In each down sampling process, the size of the image is reduced, the resolution is reduced, and the number of characteristic channels is doubled. Correspondingly, each step of deconvolution in the expansion path will reduce the number of channels by half, and copy and cross with the previously saved low-level feature map of the same scale. Then, the obtained results are sampled again, and the process is repeated until the image is restored to the original scale. This structure is also called encoder decoder structure. The contraction path corresponds to the encoder, and the expansion path corresponds to the decoder. The encoder part of U-net downsamples 4 times, and the decoder part of u-net upsamples 4 times. The feature image obtained from the down sampling of left encoder is restored to the resolution of the original image. Finally, the final output segmentation image is obtained by softmax (13).

U-net adopts splicing fusion mode, which is completely different from other common segmentation networks such as full convolution network. It stitches the features together in the dimension of channels, which is equivalent to doubling the number of channels to form thicker features. In the case of full convolution network fusion, the corresponding points are added together, and the dimensions will not change, and no thicker features will be formed.

Compared with FCN and deeplab, U-net performs four upsampling, and uses the method which connects the low-level feature map to the high-level feature map. U-net does not directly carry out the back propagation of supervision and loss function on the high-level semantic feature map, which not only ensures that the recovered feature map integrates more low-level features, but also makes the features of different scales get fusion and reference, so as to make better prediction and more fine edge information.

#### Gastric Wall Stratification Based on SRAD (Speckle Reducing Anisotropic Diffusion)

Given an image *I_0_(x,y)* with finite energy and no zero intensity value, the output image *I(x,y;t)* is obtained by the following partial differential equation:

(1) $$\{\begin{array}{c} \frac{\partial I(x,y;t)}{\partial t}=div[c(q)\nabla I(x,y;t)]\\ I(x,y;0)=I_{0}(x,y),(\frac{\partial I(x,y;t)}{\partial \vec{\Omega }})|\partial \Omega =0 \end{array}$$

It is called the *SRAD PDE*, in the same form as anisotropic diffusion. The diffusion coefficient is defined as:

(2) $$c(q)=\frac{1}{1+\frac{q^{2}(x,y;t)-{q_{0}}^{2}(t)}{{q_{0}}^{2}(t)[1+{q_{0}}^{2}(t)]}}$$

or

(3) $$c(q)=\exp[-\frac{q^{2}(x,y;t)-{q_{0}}^{2}(t)}{{q_{0}}^{2}(t)[1+{q_{0}}^{2}(t)]}]$$

The instantaneous coefficient of variation is defined as:

(4) $$q(x,y;t)=\sqrt{\frac{\frac{1}{2}{\left(\frac{\left|\nabla I\right|}{I}\right)}^{2}-\frac{1}{16}{\left(\nabla^{2}I/I\right)}^{2}}{{\left(1+\frac{1}{4}\frac{\nabla^{2}I}{I}\right)}^{2}}}$$

*q (x, y; t)* represents the degree of dispersion between pixels, which is large at the edge and small in the homogeneous region. *q_0_(t)* is a speckle scale function.

SRAD encourages isotropic diffusion in homogeneous regions, where *q* fluctuates and *C (q)* is about 1. In addition, it is necessary to manually select a homogeneous region to determine the value (14). After SRAD, the edge structure of gastric wall of the image is more clear.

#### Quantitative Measurement of Gastric Wall

After we get the image of gastric wall, because the gastric wall has five layers of staggered structure, after the SRAD anisotropic diffusion filtering processing, there will be a more obvious difference between the light and the dark, that is, the edge is strengthened by SRAD and become more obvious. In this case, the edge detection algorithm can be used to find out the junction of each layer, and the thickness of each layer is the difference of the positions of each layer. In this paper, Sobel operator is used for edge detection (15).

The following method is proposed to determine whether the patient with gastric ultrasound image has gastric disease:

When the gastric wall of one patient can be divided into five layers, the proportions for the five layers in the entire gastric wall are calculated and combined to be recorded as *x* which is a vector with 5 dimensions. Then the standard value recorded as *s* can be obtained by averaging the *x* for all the patients and we can have *s* = (0.278, 0.133, 0.154, 0.154, 0.280) in our study. The distance *d* between *s* and the *x* for one patient is defined as following

(5) $$d=\sqrt{{\left(x_{1}-s_{1}\right)}^{2}+{\left(x_{2}-s_{2}\right)}^{2}+{\left(x_{3}-s_{3}\right)}^{2}+{\left(x_{4}-s_{4}\right)}^{2}}$$

*d* is the value that we use to determine the situation of gastric wall of one patient. If the value of *d* is large, it might be regarded as abnormal gastric wall.

## Results

### Detection Results

In our study, there are three methods of labeling applied for obtaining the ground truth of gastric wall which are as following.

**Figure 4:**
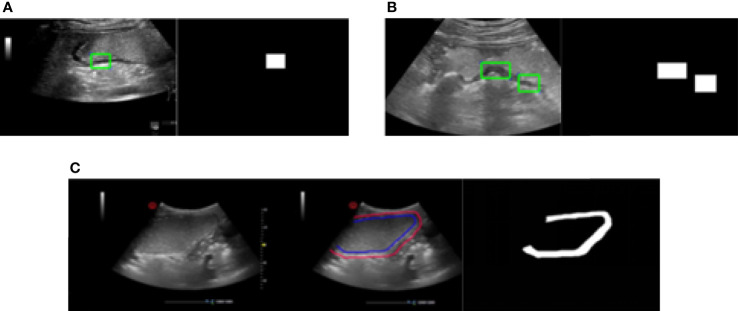
Three methods of labeling. **(A)** Apply one rectangle to label the part of the gastric wall area. **(B)** Apply two rectangles trying to cover more parts of the gastric wall area compared with the method with one rectangle. **(C)** Label the entire area of gastric wall.

1. Apply one rectangle to label the part of the gastric wall area shown as **Figure 4A**
2. Apply two rectangles trying to cover more parts of the gastric wall area compared with the method with one rectangle, which is shown in **Figure 4B**
3. Label the entire area of gastric wall shown as **Figure 4C**.

The principle of the labeling is to cover the gastric wall as much as possible. However due to the complexity of the ultrasound image representation of the gastric wall in clinical practice, it is not always possible to accurately select the entire region of gastric wall. This is the reason that we apply the above three methods for the labeling in our study. It should be denoted that the labeling results based on all of them are considered as the ground truth of gastric wall for the task of gastric wall detection.

Intersection over Union (IoU) is applied here as the metric to quantitatively evaluate the performance of the proposed model for the gastric detection, which is defined as:

(6) $$IoU=\frac{A\cap^{}B}{A\cup^{}B}$$

where A denotes the detection result and B is the ground truth of gastric wall. The symbol of ∩ denotes the intersection of the two regions and the symbol of ∪ denotes the union of the two regions. The high value of the IoU denotes the good performance of model detection. The detailed results are shown in **Table 2**.

**Table 2:** Gastric wall detection results.

Labeling method	IoU
One rectangle	0.36
Two rectangles	0.32
Label the entire area	0.43

**Figure 5:**
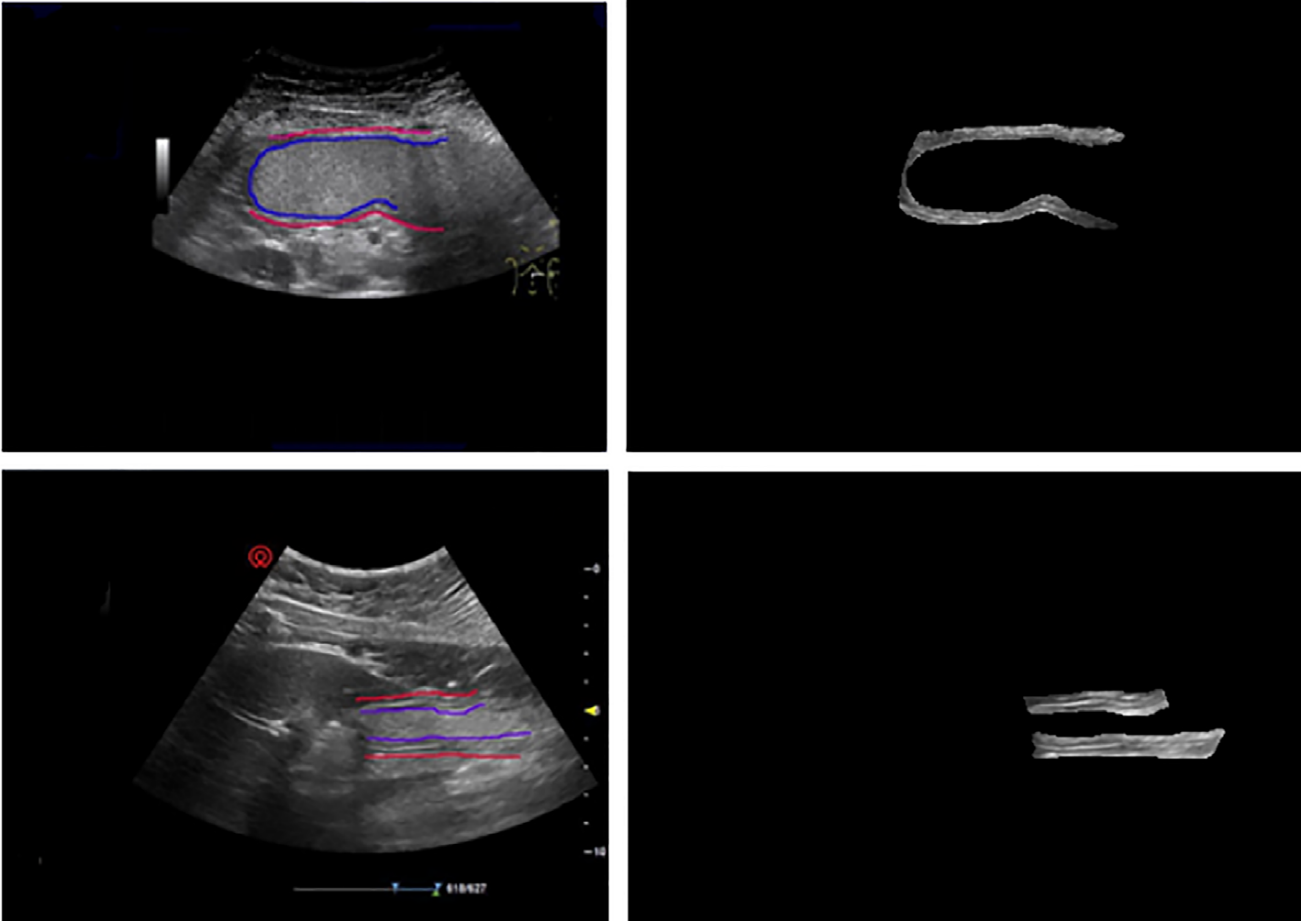
The prediction results of gold standard and our model. After training, the model can accurately predict the gastric wall area in gastric ultrasound images.

It can be seen that the proposed deep model for the detection of gastric wall demonstrates its relatively effectiveness to some extent. The fact that the largest IoU value comes from the method of labeling the entire area of gastric wall denotes that the proposed model effectively learns to represent the gastric wall. Therefore, the detection result may achieve better IoU when the ground truth is the true region of the gastric wall. It should be also noted that the performance of the detection part should be ultimately evaluated only by the results of the classification to quantitatively determine the situation of the patient as the following section since it is the only purpose of our study. **Figure 5** gives two examples of gastric wall detection based on U-net.

### Quantitative Stratification Results

**Figure 6:**
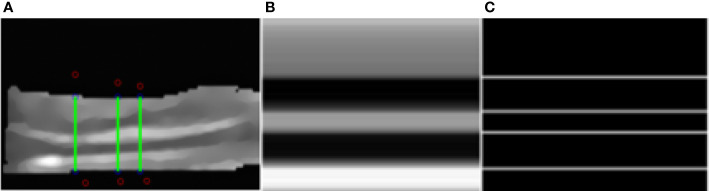
Segmentation results. **(A)** The green lines. **(B)** The pixel value of one line. **(C)** Detected edges.

We select a column in the image, corresponding to the green line in the image (**Figure 6A**). The ratio is obtained by edge detection of pixels in the column. Each line corresponds to a column of pixel values (**Figure 6B**). All the white lines in the image represent the position of the edge detected by Sobel edge detection (**Figure 6C**). Four edges of five-layer structure are detected, which is consistent with our expectation. It is easy to get the vertical position of each white line. We can calculate the relative thickness of each layer of structure and the ratio between five layers of structure by making a simple subtraction.

We select a number of such columns and average the results as the final ratio result. In this example, the proportional relationship between the five structures is 0.358: 0.189: 0.116: 0.2: 0.137.

**Figure 7:**
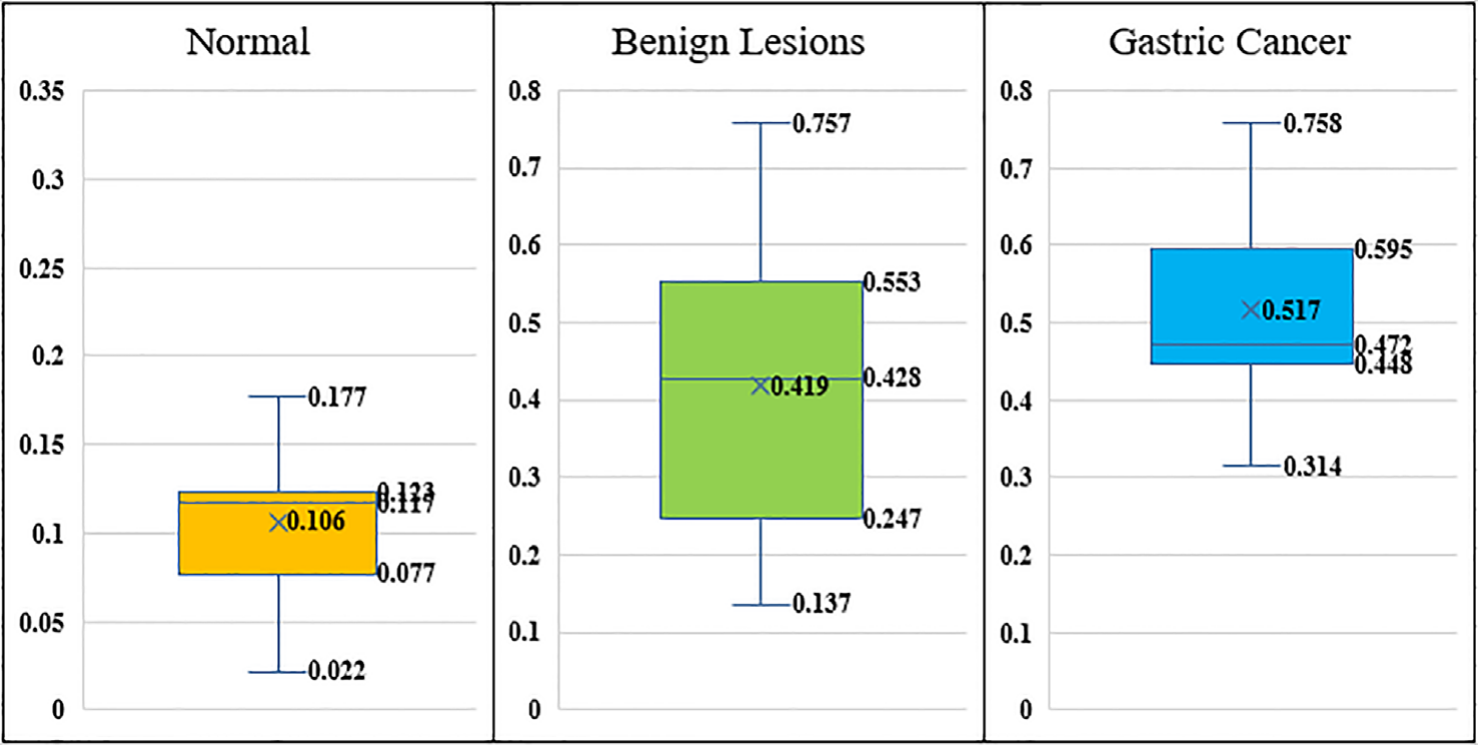
The distributions of *d* values.

For the three cohorts in our study, we calculated the *d* value defined as Eq. 5 for all the patients. **Figure 7** demonstrates the distributions of *d* values and it can be seen that the difference among normal, benign lesions and gastric cancer is quite obvious.

For the classification of gastric walls among normal, benign lesions and gastric cancer, the corresponding experiments were performed and the results are demonstrated in **Table 3**. We randomly select 70% of the data in the dataset as the training set and 30% as the test set. In the training set, we find the threshold which can best distinguish normal and abnormal cases, and apply this threshold to the test set. Results were validated by quantitative indexes including Accuracy (ACC), Sensitivity (SENS), Specificity (SPEC), Positive Predictive Value (PPV), Negative Predictive Value (NPV), Matthew’s Correlation Coefficient (MCC), F1 score and P-value between two target groups. Supposing TP, TN, FP, FN represent true positive, true negative, false positive and false negative, then accuracy (ACC), sensitivity (SENS), specificity (SPEC), positive predictive value (PPV), negative predictive value (NPV), Matthew’s correlation coefficient (MCC) and F1 score can be defined and calculated as:

**Table 3:** Classification results based on diagnostic model.

Experiment	AUC	ACC	SENS	SPEC	PPV	NPV	MCC	F1score	P-value
Normal vs Benign Lesions	0.90	0.94	1.00	0.80	0.93	1.00	0.86	0.96	<0.0001
Normal vs Gastric Cancer	0.93	0.92	1.00	0.86	0.83	1.00	0.85	0.91	<0.0001
Normal vs Benign Lesions & Gastric Cancer	0.92	0.95	1.00	0.83	0.93	1.00	0.88	0.97	<0.0001

$$ACC=\frac{TP+TN}{TP+TN+FP+FN}$$

$$SENS=\frac{TP}{TP+FN}$$

$$SPEC=\frac{TN}{TN+FP}$$

$$PPV=\frac{TP}{TP+FP}$$

$$NPV=\frac{TN}{TN+FN}$$

$$MCC=\frac{TP\times TN-FP\times FN}{\sqrt{\left(TP+FP\right)\left(TP+FN\right)\left(TN+FP\right)\left(TN+FN\right)}}$$

$$F_{1}=2\cdot \frac{Precision\cdot Recall}{Precision+Recall}$$

It can be seen that the proposed quantitative method achieves excellent results for the classification of gastric walls among normal, benign lesions and gastric cancer.

## Conclusions and Discussion

The main method for gastric cancer diagnosis and screening in developed countries is gastroscopy, which has significantly increased the early detection rate of gastric cancer and improved the survival rate of patients. Gastroscopy and gastric mucosal biopsy techniques are also highly recommended in China. However, the early diagnosis rate of gastric cancer is still very poor, with the early diagnosis and treatment rate of gastric cancer is only about 10%. China is a developing country with more than half of the rural population. Not only is there an inherent causal relationship between certain traditional eating habits of rural residents and the occurrence of gastric cancer, but the low self-care awareness of rural population is a relatively high-risk factor for gastric cancer. What is even more unfavorable is that the number of physicians who can perform gastroscopy in rural areas is severely insufficient, and rural residents have low compliance with gastroscopy, resulting in serious practical difficulties in gastroscopy screening for gastric cancer (16). Therefore, it is urgent to explore and establish a gastric cancer screening and diagnosis strategy with Chinese characteristics.

Among many clinical imaging techniques, ultrasound imaging has the advantages of high soft tissue resolution, easy operation, safety and painlessness. In China, the penetration rate of ultrasound equipment is extremely high, and ultrasound equipment at all types of medical institutions must be equipped. For gastric cancer, ultrasound can show the location, size, level of invasion of the stomach wall, and whether there are swollen lymph nodes in the stomach.

The stomach is a hollow organ when without filling, so it is indeed hard to obtain accurate measurement of gastric wall structure by ultrasound. Therefore, we used oral contrast trans-abdominal ultrasonography, which has obvious advantages. The contrast agent fills the stomach cavity to form a high-quality “acoustic window”, which is effective in improving the ultrasound imaging ability of the stomach wall structure. For most of the conventional ultrasound diagnostic apparatuses, the imaging quality can improve the hierarchical structure and continuity of the stomach wall, and the recognition ability can meet the detection of most gastric cancer.

We have proposed a U-net based model for automatic recognition of gastric wall region from gastric ultrasound images. We use speckle reduced anisotropic diffusion to make the hierarchical structure of gastric wall more obvious. By dividing the five layers of gastric wall and calculating the ratio of each layer, the normal gastric wall and abnormal gastric wall can be accurately distinguished. This method is also a useful attempt for early screening of gastric cancer. In the existing cases, the detection accuracy of abnormal gastric wall is 95%.

As far as we know, there is no quantitative analysis of the thickness of five layers of gastric wall before. This article is a preliminary report on this aspect. In normal and abnormal cases, the ratio of gastric wall thickness is different. The change of gastric wall thickness often means the occurrence of gastric diseases, and our experimental results have confirmed it. The experimental results have shown that the proposed detection and calculation method of gastric wall is helpful to quantify the diagnosis information of gastric diseases, and is expected to improve the efficiency of ultrasound screening for gastric cancer.

In the future, with the advantages of noninvasive safety, cost-effectiveness, high equipment penetration rate and inspection compliance, gastric ultrasound will play more unique roles and advantages in the diagnosis and screening of gastric cancer.

## Data Availability Statement

The raw data supporting the conclusions of this article will be made available by the authors, without undue reservation.

## Ethics Statement

The studies involving human participants were reviewed and approved by Ethic Committee of Xinhua Hospital Affiliated to Shanghai Jiaotong University. Written informed consent to participate in this study was provided by the participants’ legal guardian/next of kin.

## Author Contributions

AS and ZH wrote the article. YD provided the technical guidance. AS and XX made the Figure. YW has made substantial contribution to the work. JY and LS conceived and designed the idea. All authors contributed to the article and approved the submitted version.

## Funding

This work was supported by Shanghai science and technology action innovation plan (19441903100).

## Conflict of Interest

The authors declare that the research was conducted in the absence of any commercial or financial relationships that could be construed as a potential conflict of interest.
